# Porcelain Bodies, Colors and Shadows

**Published:** 1901-05-15

**Authors:** W. S. Taggart


### PORCELAIN BODIES, COLORS AND SHADOWS.

BY W. S. TAGGART, D.D.S.

While bodies, colors and shadows may play an essential part in this artistic process, I do not think that either the porcelain bodies or colors will ever be furnished us that can make porcelain inlay work artistic. The artistic effect must come from ability to choose the proper porcelain for any particular case. Do not put off learning this process because you think some day you will have perfect materials and then you will commence, but commence now and by the time you have acquired a certain amount of ability, you will appreciate the fact that it is the "know how" part that is more essential than the materials.

There are certain underlying principles that must not be lost sight of. An inlay can never be made to match a tooth when only one color is used in its construction. Take, for instance, an approximal cavity in a central incisor extending any part of the distance from the gum margin to the incisal edge. No matter how perfectly you may match either the incisal edge, which is one color, or the neck of the tooth, which is another color, the inlay will look badly. If the neck were made of yellow porcelain and the tip with blue porcelain or whatever color was necessary to match it, and the two colors given a gradual gradation by blending one into the other, the effect would be very artistic. The main reason a cement filling in a front tooth looks so inartistic is because it is all of one color and yet this cement may match beautifully either the tip or neck, but can not match both. So I say, to get correct effects, blend your colors.

Another principle, you can get much better effects if the bulk of your porcelain is made of the yellow porcelains and the colors to match the tooth put on top of this, in this way you get the soft blending of the colors and the rays of light do not pass as they would through a piece of glass or a piece of porcelain, all of one color. If you examine closely a natural tooth or a beautifully colored artificial tooth you will almost always find the yellows predominate on the palatine side. Another thing that will affect the color of a porcelain inlay is the thickness of it. Supposing the bulk of the filling is made of yellowish tinge and you choose a certain color of porcelain to veneer over this to match the tooth, you will find you can get several different shades according to the thickness of the porcelain, and here again comes in the advantage of the "know how" part.

Another thing that will affect the shade of a given color of porcelain is the way in which it is packed.

One dentist who condenses his porcelain thoroughly before it is baked will get a porcelain several shades darker than one who carelessly packs the porcelain, for the reason that air spaces in a given color of porcelain have a tendency to lighten the shade.

Shall it be the high fusing requiring a platinum matrix or the low fusing using a gold matrix? My preference has been and is now in favor of the high fusing. Why? Because it seems to take a more natural or rather vital coloring. The low fusing porcelains get their color from the pigments mixed with them, the same as we color white lead paint by mixing so much of any coloring matter with it; whereas the coloring in high fusing porcelains is brought out by the chemical changes which take place at the high temperatures necessary to fuse it, and this gives the more life-like appearance to the porcelain. The lower fusing, while made in a great variety of colors which we can apparently match, always seem to have a muddy, opaque look as compared to the vital, translucent look of high fusing porcelains.

Shall we use inlays or not? Yes, but not indiscriminately. Choose cases and only put them where the necessity for artistic effects outweighs all others. Not but what they are lasting and satisfactory in saving the tooth from decay, but for every other quality except looks, nothing is better than gold as a filling material. Remember that in making these fillings the results you get will depend on your ability to handle the material and not on the material itself, bearing in mind that some dentists can put in good gold fillings with any gold made, while others can not put in good gold fillings with the best gold that was ever manufactured.

But do not be discouraged about your first results. Remember it is a new line to you and will require a patience and perseverance and skill which no other part of dentistry requires, but when you once begin to get satisfactory results you will be amply repaid for your efforts.—*March Dental Review.*

